# The 45-month therapy outcomes of permanent seed implantation and radical prostatectomy for prostate cancer patients

**DOI:** 10.1007/s10637-021-01189-0

**Published:** 2022-02-12

**Authors:** Chao Li, Mengdong Zhang, Jianwen Wang, Xiaodong Zhang

**Affiliations:** 1 Department of Urology Surgery, Shijiazhuang People´s Hospital, 36 Fanxi Road, 050011 Shijiazhuang, China; 2 Urology Institute of Capital Medical University, Department of Urology, Beijing ChaoYang Hospital, Capital Medical University, No. 8 Gong Ti Nan Lu, 100020 Beijing, China

**Keywords:** Permanent seed implantation, Radical prostatectomy, Survival rate, Security, Efficacy

## Abstract

*Objective* To compare the efficacy and safety-related outcomes after radical prostatectomy (RP) and permanent seed implantation (PI) using iodine-125 seeds in patients with prostate cancer. *Method* A retrospective analysis of 196 patients with biopsy-confirmed prostate cancer (T2-T3) was performed in this study. Forty-five patients who underwent PI using iodine-125 seeds combined with endocrine therapy or androgen deprivation therapy (ADT) were compared with 151 patients who underwent RP combined with endocrine therapy or adjuvant ADT. The efficacy and safety outcomes were compared using Kaplan–Meier curves and *t*-tests. *Results* Between the RP and PI treatment modalities, no significant difference (*P* > 0.05) in biochemical recurrence-free survival (BRFS) was observed using Kaplan–Meier curves, regardless of the combination of adjuvant treatment modalities. Furthermore, no significant differences were observed (*P* > 0.05) with respect to PSA fluctuations, albumin, leukocyte count, urinary and rectal symptoms, erectile function or quality of life (QoL) between the two therapy methods. However, significant differences in the maximum flow rate, average length of hospital stay and indwelling catheter time were observed between the two groups (*P* < 0.001). *Conclusion* Iodine-125 seed implantation significantly shortened the average length of hospital stay and indwelling catheter time compared with RP, and the haemoglobin level was significantly higher in the PI group than in the RP group; however, the maximum urine flow rate was lower after of PI than after RP. These two methods showed similar BRFS rates among prostate cancer patients.

## Introduction

Prostate cancer is the most common cancer among males, and the incidence is in increasing [1]. By screening PSA levels, prostate tumours confined to the prostate gland can be diagnosed at an early stage. Prostate cancer patients face a confusing choice between radical prostatectomy (RP) and brachytherapy. Radical prostatectomy is considered to be the gold standard and an effective therapy choice for localized prostate cancer [2]. The most popular technique for brachytherapy is permanent seed implantation, and the most commonly used isotope is iodine-125[3]. Brachytherapy has been shown to be relatively minimally invasive, lead to a reduced morbidity and is associated with a minimal length of hospital stay[4]; furthermore, more than 80% of patients are free from biochemical recurrence within 10 years[5]. A large amount of data from the United States also showed that brachytherapy with permanent seeds was an effective treatment for patients with localized prostate cancer[6]. Previous reports [3, 7] also suggested that RP and brachytherapy treatments were equivalent in biochemical control; however, a comparison safety between these two methods remains to be performed. In the present study, we aimed to compare BRFS and safety-related parameters in prostate cancer patients treated with RP or PI at a single hospital.

## Materials and methods

### Patients

From November 2013 to January 2017, 196 patients who were diagnosed pathologically with prostate cancer through transrectal ultrasound-guided biopsies of the prostate (T2-3) at our institution were enrolled in this study. All patients in this study were consecutive patients. None of the patients had other urinary system diseases or prior operations. Forty-five patients underwent PI, and 151 patients were treated with RP; the choice of therapy type was made by both the doctor and the patient. All RP and PI treatments were performed by the same surgeon. Patients without previous treatment, follow-up PSA levels and Gleason scores were excluded from this study. Distant metastasis and lymphatic metastasis cases were also excluded from this study. In the PI group, 15 patients also received endocrine therapy preoperatively, and 13 received ADT therapy postoperatively, while 52 and 44 patients received endocrine therapy and ADT therapy in the RP group, respectively. All data were obtained from a prospectively maintained database. All patients enrolled in this study provided signed informed consent forms, and our research was approved by the ethics committee of our institution.

All patients were evaluated based on medical history, biopsies of the prostate using transrectal ultrasound guidance, pre-treatment PSA level (iPSA), digital rectal examinations, bone scans using computed tomography and serum chemistry examinations.

### PI and RP therapies

In the present study, the PI therapy procedures were similar to those in previous reports[3, 6]. Briefly, the dimensions of each prostate were measured through transrectal ultrasonography pre brachytherapy to confirm the overall isotope activity required for each patient, and the number of seeds required for implantation was calculated by dividing the overall activity required by the activity of each seed at the implant point. The target area was a 5-mm margin around the lateral and anterior prostate. The seeds were then introduced to their target positions by the radiation oncologist and urologist. The radical prostatectomy procedures were similar to the approach used in previous reports [3, 8].

### The efficacy and safety outcomes after each therapy

Patients who underwent RP or PI were monitored with serum PSA levels at 1, 6 and 12 months after treatment and further evaluated at 24 and 36 months in the second year of follow-up. Particle transfer, maximum urine flow rate, International Index of Erectile Function (IIEF), rectal symptoms, postoperative length of hospital stay and recovery of urinary control were used to assess the safety of the RP and PI treatments.

### Statistical analysis

IBM SPSS 19.0 software was used for statistical analysis. Comparisons between the two therapy modalities pre and post treatment were performed using *t*-tests for continuous variables and chi-square tests for enumeration data. *P* < 0.05 was considered statistically significant. Survival probabilities were determined by using the Kaplan–Meier curve, and the survival estimated for prostate cancer patients was determined according to the therapy type (IP vs RP) and use of neoadjuvant endocrine therapy or ADT therapy (yes or no). The survival probability differences between the two curves were analysed by the log-rank test.

## Results

The baseline characteristics of the prostate cancer patients in the present research are shown in Tables 1 and 2. The patients who underwent permanent seed implantation had an age range of 58–85 years (mean 74.8 ± 6.3), while the patients treated with radical prostatectomy were aged 27–87 years, with a mean of 72.5 ± 7.9 years (*P* = 0.25). No significant differences were observed with respect to BMI, iPSA, Gleason score, prostate volume, maximum tumour load or clinical stage between the two cohorts (*P* > 0.05). Furthermore, the results of conventional physical examinations, including bone scan, digital rectal examination, blood pressure, blood sugar and adjuvant treatment using ADT and endocrine therapy, showed no significant differences between these two groups (*P* > 0.05).

**Table 1:** Baseline characteristics

	PI (N = 45)	RP (N = 151)	*P*-value
Mean age ± SD (years)	74.8 ± 6.3 (58–85)	72.5 ± 7.9 (27–87)	0.25
Mean BMI (kg/m^2^)	24.8 ± 3.1	24.3 ± 3.3	0.18
iPSA (ug/ml)	21.6 ± 18.1	24.4 ± 10.1	0.60
Mean prostate volume ± SD (ml)	48.3 ± 23.5	45.2 ± 17.1	0.43
Mean Gleason score	7.5 ± 1.1	7.6 ± 2.1	0.74
Number of positive needles	2.5 ± 1.5	2.7 ± 1.8	0.84
Maximum tumour load (%)	39.2 ± 19.0	41.2 ± 14.1	0.66

PI and RP represent permanent seed implantation and radical prostatectomy, respectively. *P* < 0.05 represent a significant difference between the PI and RP groups

**Table 2:** Main clinical parameters of the physical examination

	PI n (%)	RP n (%)	*P*-value
Clinical stage			0.34
2a	8 (17.8)	16 (10.6)	
2b	8 (17.8)	39 (25.8)	
2c	17 (37.8)	42 (27.8)	
3a	11 (24.4)	47 (31.1)	
3b	1 (2.2)	7 (4.7)	
Bone scintigraphy			0.67
Normal	31 (68.9)	109 (72.2)	
Abnormal	14 (31.1)	42 (27.8)	
Hypertension			0.28
No	35 (77.8)	105 (69.5)	
Yes	10 (22.2)	46 (30.5)	
Diabetes mellitus			0.19
No	35 (77.8)	102 (67.5)	
Yes	10 (22.2)	49 (32.5)	
Neoadjuvant endocrine therapy			0.89
No	30 (66.7)	99 (65.6)	
Yes	15 (33.3)	52 (34.4)	
ADT therapy			0.97
No	32 (71.1)	107 (70.9)	
Yes	13 (28.9)	44 (29.1)	
Digital rectal examination			0.46
Negative	16 (35.6)	63 (41.7)	
Positive	29 (64.4)	88 (58.3)	
MRI			0.91
Negative	12 (26.7)	39 (25.8)	
Positive	33 (73.3)	112 (74.2)	

PI and RP represent permanent seed implantation and radical prostatectomy, respectively. MRI indicates magnetic resonance imaging. *P* < 0.05 represents a significant difference between the PI and RP groups

**Fig. 1:**
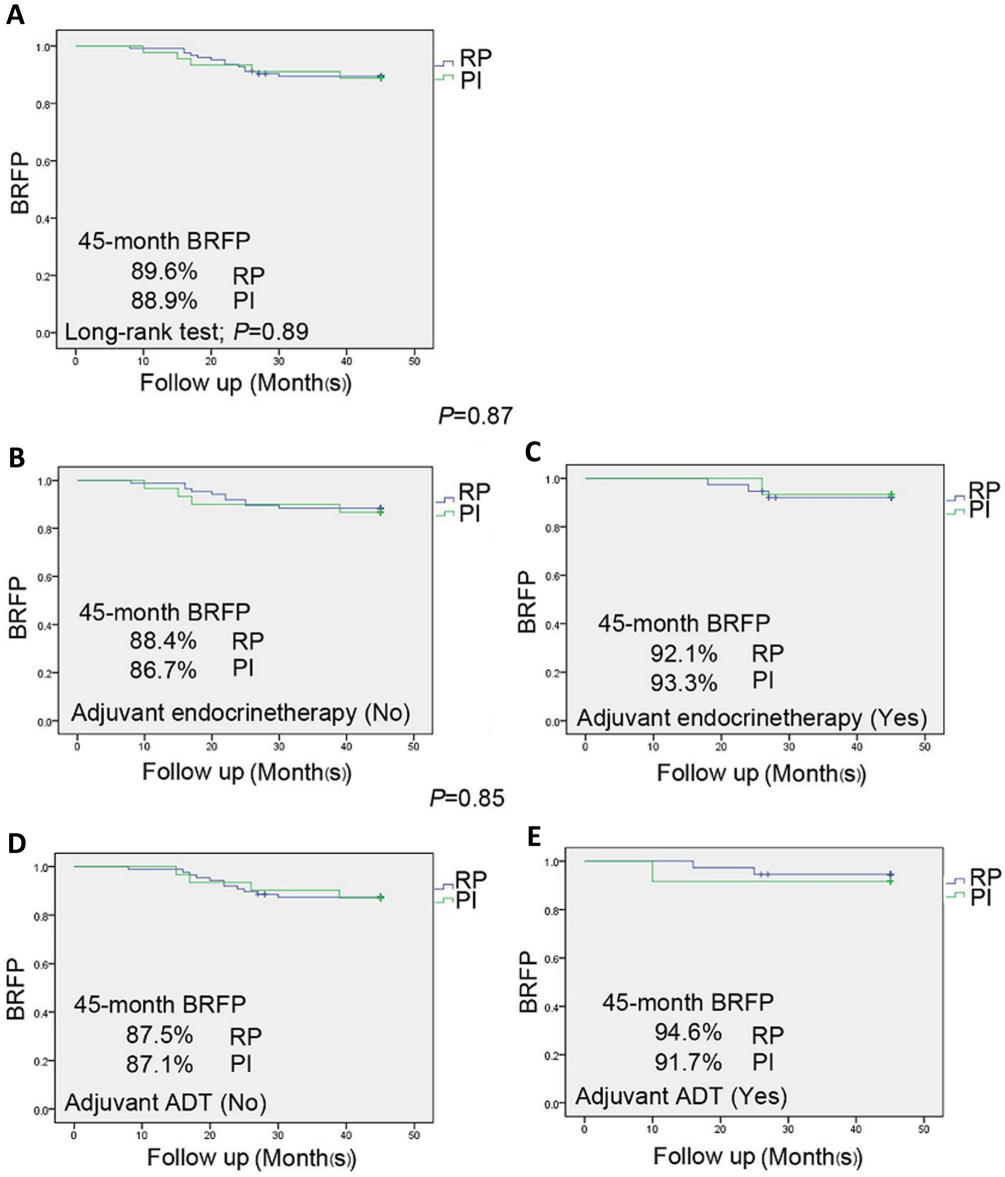
Kaplan–Meier estimates of biochemical relapse-free survival according to treatment modality for patients with prostate cancer. PI, permanent seed implantation; RP, radical prostatectomy. **A** BRFS over a 45-month period for the entire cohort that underwent PI (45 patients) and RP (151 patients); *P* = 0.89, representing no significant difference. **B** and **C** Survival curves for the entire cohort with **B** and without **C** adjuvant endocrine therapy. **D** and **E** Survival curves for the entire cohort with **D** and without **E** adjuvant ADT treatment. PI, RP, ADT represent permanent seed implantation, radical prostatectomy and androgen deprivation therapy, respectively. *P* = 0.87 and *P* = 0.85 represent no significant differences in BRFS between the PI and RP groups stratified by adjuvant endocrine therapy and ADT, respectively

**Fig. 2:**
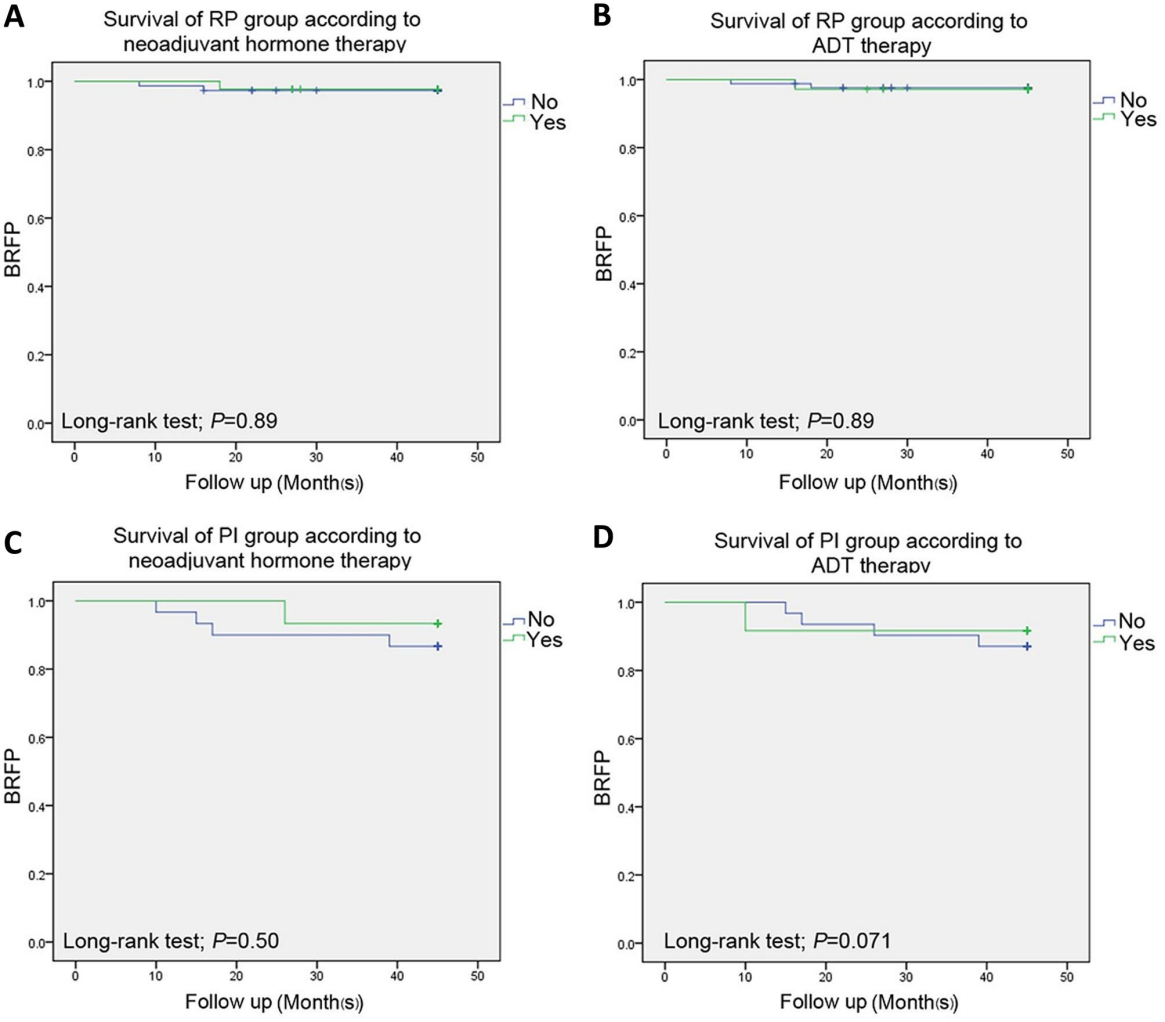
Kaplan–Meier estimates of biochemical relapse-free survival of prostate cancer patients treated with RP **A** and **B** and PI **C** and **D** with neoadjuvant hormone/ADT therapy or not. PI, permanent seed implantation; RP, radical prostatectomy. *P* > 0.05 represents no significant difference between the prostate patients with and without adjuvant treatments

**Fig. 3:**
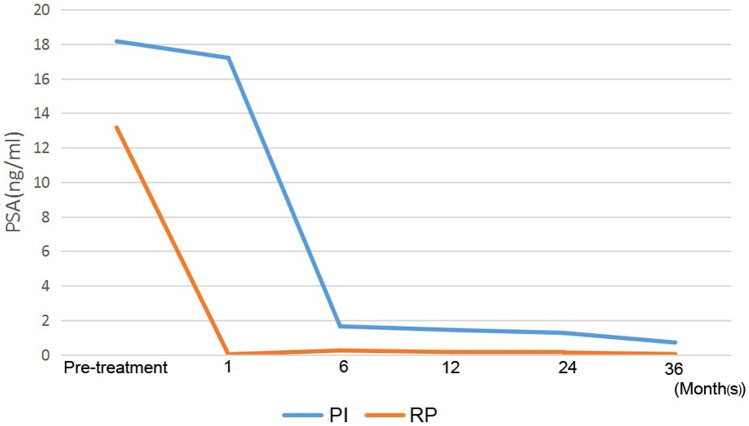
Cancer control was determined by screening PSA levels. The PSA levels continued to decrease for three years after PI and RP in this study. PI and RP represent permanent seed implantation and radical prostatectomy, respectively

For the PI and RP treatment groups, the 45-month biochemical recurrence survival rates were 89.6% and 88.9%, respectively, and no statistically significant difference (*P* = 0.89) existed between the two groups according to the Kaplan–Meier analysis (Fig. 1A). When stratifying survival by the use of adjuvant endocrine therapy (yes vs. no), the log-rank analysis revealed no statistically significant difference in BRFS between the PI and RP treatments (*P* = 0.87) (Fig. 1B-C). In the univariate model, the use of ADT postoperatively (yes vs. no) was treated as a dichotomous variable, and the Kaplan–Meier analysis showed that there was no significant difference between the PI and RP treatments with respect to BRFS (*P* = 0.85) (Fig. 1D-E). Prostate cancer control after PI and RP was monitored by evaluating serum PSA fluctuations at 1, 6, 12, 24 and 36 months of follow-up. The biochemical recurrence-free survival rates of the patients with and without adjuvant therapies were further analyzed, and the log-rank test showed no statistically significant difference was observed between the prostate cancer patients with and without neoadjuvant hormone/ADP therapies in RP group (*P* = 0.89) (Fig. 2A and B), between the PI treated patients with and without neoadjuvant hormone/ADP therapies (*P* = 0.50 and *P* = 0.071), respectively (Fig. 2C and D). As shown in Fig. 3, the patients in both the PI and RP groups had a continuous reduction in PSA postoperatively. Three years after PI treatment, the PSA levels decreased from 18.2 to 0.7 ng/ml.

The PI group had a significantly lower maximum urine flow rate, shorter average length of hospital stay and shorter indwelling catheterization time than those in the RP group (*P* < 0.05), and the haemoglobin level was significantly higher in the PI group than in the RP group (*P* < 0.05). However, no significant difference existed between the two treatments with respect to WBC count, albumin and haemoglobin level (P > 0.05). Furthermore, the QoL and IIEF scores were 52.0 ± 5.6 and 23.4 ± 1.2 in the PI group and 50.7 ± 6.2 and 21.2 ± 3.5 in the RP group, respectively, and no significant difference was found between these two treatments (P > 0.05) (Table 3).

**Table 3:** Safety-related parameters of patients who underwent PI and RP

Postoperative variables	PI (N = 45)	RP (N = 151)	*P*-value
Maximum urine flow rate (ml/s)	14.7 ± 3.4	23.4 ± 2.0	0
Average length of hospital stay (days)	3.7 ± 1.5	7.7 ± 3.5	0
Indwelling catheterization (days)	3.2 ± 1.2	10.4 ± 6.2	0
QoL	52.0 ± 5.6	50.7 ± 6.2	0.84
IIEF score	23.4 ± 1.2	21.2 ± 3.5	0.61
Haemoglobin (g/L)	112.0 ± 24.1	90.2 ± 18.3	0.02
WBC (^*^10^9^)	6.3 ± 1.3	7.8 ± 2.3	0.1
Albumin (g/L)	41.3 ± 7.2	36.2 ± 9.2	0.26

PI and RP represent permanent seed implantation and radical prostatectomy, respectively. QoL and WBC indicate quality of life and white blood cell, respectively

## Discussion

Radical prostatectomy is the gold standard therapy for localized prostate cancer because of its superior cure rate[7]. With the development of radiation therapy and radiological technology, the positive outcomes after permanent seed implantation have improved in recent years compared with previous reports[9]. Many studies have compared the BRFS rates of prostate cancer patients treated with RP, PI and external-beam radiotherapy. However, safety-related outcomes remain to be established. In this study, we compared the efficacy and safety of radical prostatectomy and permanent seed implantation, including pain relief, psychological and physiological burden and degree of satisfaction with the therapeutic effects.

It was reported that the survival rate of patients treated with brachytherapy (79.7%) was higher than that of patients treated with radical prostatectomy (44.3%)[3]; however, many previous studies[7, 10] also showed that the biochemical failure rates between radical prostatectomy and permanent prostate brachytherapy were similar. Our results revealed that the biochemical recurrence-free survival of all patients between the two therapies was similar, with no significant difference (*P* > 0.05). The effects of brachytherapy on prostate cancer were heterogeneous and were possibly associated with brachytherapy technique and differences in methodology to compare the outcomes of surgery and brachytherapy. It was also reported that biochemical recurrence-free survival was determined more by the intrinsic characteristics of the tumour than by a specific therapy modality at the time of treatment [11]; thus, the tumour characteristics before therapy are associated with the treatment outcomes.

It was reported that the combination of multimodality treatment, including ADT, showed a higher progression-free survival than seed implantation or surgery alone for prostate cancer patients [7]. In the present study, 15 and 13 patients were treated with PI therapy combined with neoadjuvant endocrine therapy or ADT, respectively, and 52 and 44 patients underwent RP therapy combined with neoadjuvant endocrine therapy or ADT, respectively. Our results showed similar Kaplan–Meier curves for BRFS in prostate cancer patients treated with PI or RP combined with or without neoadjuvant endocrine therapy or ADT, indicating that no significant differences (*P* > 0.05) in BRFS were observed between the PI and RP groups, regardless of treatment with monotherapy alone or combined adjuvant therapies.

A comparison of the safety-related outcomes after radical prostatectomy and permanent seed implantation using iodine-125 seed groups has not been performed. Tanake et al. [12] analysed acute and late genitourinary (GU) toxicity in prostate cancer patients who underwent PI therapy and revealed that PI therapy alone induced a significantly higher rate of GU toxicity than PI combined with external-beam radiation therapy. The acute effects of permanent brachytherapy on the urinary tract include urge incontinence, urgency of urination, haematuria, painful micturition and urinary retention [13]. In line with previous studies, our results also showed that the maximum urine flow rate in the seed implantation group was 14.7 ± 3.4 ml/s, which was significantly lower than that after radical prostatectomy treatment (23.4 ± 2.0 ml/s) (*P* < 0.05), which may be because gland retention and tissue oedema after seed implantation are associated with the maximum urine flow rate.

In this study, the average length of hospital stay and indwelling catheterization time of patients who underwent permanent seed implantation were 3.7 ± 1.5 and 3.2 ± 1.2 days, respectively, which were significantly shorter than those in patients who underwent radical prostatectomy (7.7 ± 3.5 and 10.4 ± 6.2 days, respectively) (*P* < 0.001), indicating that the recovery period of seed implantation was shorter than that of radical prostatectomy.

Currently, the clinical curative evaluation is not limited to efficiency, safety and pain relief rate but rather includes a comprehensive patient evaluation, including satisfaction degree, psychological and mental state, as well as recovery of social functions[14]. Thus, the postoperative quality of life in patients treated with seed implantation and radical prostatectomy was studied using the QoL score in this study, and our results showed that no significant difference existed between the seed implantation and radical prostatectomy groups (*P* > 0.05), which was not in line with previous reports[7, 15] that revealed that the quality of life after seed implantation was better than that after radical prostatectomy. In our future research, we aim to determine the incidence of complications after radical prostatectomy and permanent seed implantation treatments with a larger cohort of patients. Erectile dysfunction is a therapy-induced morbidity; Merrick et al.[16] evaluated erectile function after permanent brachytherapy using IIEF and showed that the incidence rate of erectile dysfunction was 52% at 6 years, and the potency rate was 55% at 2 years according to a telephone-administered questionnaire by Chaikin et al. [17]. In the present study, we compared the IIEF scores between the permanent seed implantation (23.4 ± 1.2) and radical prostatectomy groups (21.2 ± 3.5), and no significant difference was observed between these two treatments (*P* > 0.05), indicating that the two treatments led to similar sexual function impairment.

To the best of our knowledge, few studies have compared haemoglobin, leucocyte and albumin levels postoperatively between permanent seed implantation and radical prostatectomy groups, as we have in this study. Our results showed that no significant difference (*P* > 0.05) in leucocyte and albumin existed between the two groups after treatments. However, the average haemoglobin level after radical prostatectomy was 90.2 ± 18.3, which was significantly lower (*P* < 0.001) than that after seed implantation (112.0 ± 24.1). Haemoglobin level will be studied in our future study with longer postoperative follow-up.

## Conclusions

In prostate cancer patients, permanent seed implantation using iodine-125 seeds resulted in similar biochemical recurrence-free survival to radical prostatectomy, regardless of the use of adjuvant multimodality treatments. However, the safety-related physiological parameters revealed significant differences between the two therapy methods.

## Data Availability

The datasets used and/or analysed during the current study are available from the corresponding author on reasonable request.
