# Author Correction: The 45-month therapy outcomes of permanent seed implantation and radical prostatectomy for prostate cancer patients

**DOI:** 10.1007/s10637-022-01273-z

**Published:** 2022-07-05

**Authors:** Chao Li, Mengdong Zhang, Jianwen Wang, Xiaodong Zhang

**Affiliations:** 1 Urology Institute of Capital Medical University, Department of Urology, Beijing ChaoYang Hospital, Capital Medical University, No. 8 Gong Ti Nan Lu, Beijing, 100020 China; 2 Department of Urology Surgery, Shijiazhuang People´s Hospital, 36 Fanxi Road, Shijiazhuang, 050011 China

## Author Correction : Investigational New Drugs (2022) 40:660–667 https://doi.org/10.1007/s10637-021-01189-0

Corrections are needed to the author's affiliations.

The correct affiliations are now indicated in this erratum article.

